# Simulating lightning NO production in CMAQv5.2: evolution of scientific updates

**DOI:** 10.5194/gmd-12-3071-2019

**Published:** 2019-07-18

**Authors:** Daiwen Kang, Kenneth E. Pickering, Dale J. Allen, Kristen M. Foley, David C. Wong, Rohit Mathur, Shawn J. Roselle

**Affiliations:** 1 National Exposure Research Laboratory, U.S. Environmental Protection Agency, Research Triangle Park, NC 27711, USA; 2 Department of Atmospheric and Oceanic Science, University of Maryland, College Park, MD, USA

## Abstract

This work describes the lightning nitric oxide (LNO) production schemes in the Community Multiscale Air Quality (CMAQ) model. We first document the existing LNO production scheme and vertical distribution algorithm. We then describe updates that were made to the scheme originally based on monthly National Lightning Detection Network (mNLDN) observations. The updated scheme uses hourly NLDN (hNLDN) observations. These NLDN-based schemes are good for retrospective model applications when historical lightning data are available. For applications when observed data are not available (i.e., air quality forecasts and climate studies that assume similar climate conditions), we have developed a scheme that is based on linear and log-linear parameters derived from regression of multiyear historical NLDN (pNLDN) observations and meteorological model simulations. Preliminary assessment for total column LNO production reveals that the mNLDN scheme overestimates LNO by over 40% during summer months compared with the updated hNLDN scheme that reflects the observed lightning activity more faithfully in time and space. The pNLDN performance varies with year, but it generally produced LNO columns that are comparable to hNLDN and mNLDN, and in most cases it outperformed mNLDN. Thus, when no observed lightning data are available, pNLDN can provide reasonable estimates of LNO emissions over time and space for this important natural NO source that influences air quality regulations.

## 1 Introduction

Lightning nitrogen oxide (LNO) is produced by the intense heating of air molecules during a lightning discharge and subsequent rapid cooling of the hot lightning channel (Chameides, 1986). Since NO and NO_2_ are often coexistent in equilibrium after immediate release, they are often collectively referred to as nitrogen oxides (NO_*x*_; NO_*x*_=NO + NO_2_). NO_*x*_ produced by lightning flashes is referred to as lightning NO_*x*_ (LNO_*x*_) in the literature. As one of the major natural sources of NO, LNO is mainly produced in the middle and upper troposphere. It plays an essential role in regulating ozone (O_3_) mixing ratios and influences the oxidizing capacity of the troposphere (Murray, 2016). Despite much effort in both observing and modeling LNO during the past decade, considerable uncertainties still exist with the quantification of LNO production and distribution in the troposphere (Ott et al., 2010). Most estimates of global LNO_*x*_ production range from 2 to 8 Tg N yr^−1^, which is 10%–15% of the total NO_*x*_ budget (Schumann and Huntrieser, 2007). However, owing to the concerted efforts to reduce anthropogenic NO_*x*_ emissions within the US in recent decades, it is expected that the relative burden of LNO_*x*_ and its associated impact on atmospheric chemistry will increase. As a result, it is important to include LNO_*x*_ even when modeling ground-level air quality and the interaction of air–surface exchange processes.

To simulate the amount of LNO production in space and time in a chemical transport model (CTM), it is important to know the following: (1) where and when lightning flashes occur, (2) the amount of LNO produced per flash and (3) how LNO is vertically distributed. Historically, the lightning flash rates are derived with the aid of parameterizations in CTMs (Price and Rind, 1992; Allen et al., 2000, 2010, 2012; Barthe et al., 2007; Miyazaki et al., 2014). Various schemes have been developed for determining LNO production per flash based on assumptions regarding LNO production efficiency per flash or the energy ratio of cloud-to-ground (CG) flashes to intra-cloud (IC) flashes (Schumann and Huntrieser, 2007). The derived parameterizations based on theoretical analysis (e.g., Price et al., 1997), laboratory studies (Wang et al., 1998), limited aircraft or satellite observations, or a combination of these methods, are generally too simplified, have large uncertainties (Miyazaki et. al., 2014) and cannot represent well the regional and temporal variability of lightning activity (Boccippio et al., 2000; Medici et al., 2017). Over the past decades, our understanding of the production and distribution of LNO has been greatly improved with the aid of ground-based lightning detection networks (e.g., Nag et al., 2014; Rodger et al., 2006), aircraft measurements for specific storms (e.g., Huntrieser et al., 2011), satellite observations (Pickering et al., 2016; Medici et al., 2017; Boersma et. al., 2005), and modeling studies (e.g., Zoghzoghy et al., 2015; Cummings et al., 2013). Even though there are still substantial sources of uncertainty, the LNO production rate per flash is now more robust than earlier literature estimates (Bucsela et al., 2010; Huntrieser et al., 2009, 2011; Pickering et al., 2016; Ott et al., 2010).

An LNO production module, based on the lightning flash rate and LNO parameterizations of Allen et al. (2012), was first introduced in the Community Multiscale Air Quality (CMAQ) (Byun and Schere, 2006) model Version 5.0 (CMAQv5.0) that was released in 2012. That scheme, like the schemes used in previous work (Kaynak et al., 2008; Smith and Mueller, 2010; Koo et al., 2010), uses flash rates from the National Lightning Detection Network (NLDN) (Orville et al., 2002) to constrain LNO. Specifically, LNO production is proportional to convective precipitation and is scaled locally so that the monthly average convective-precipitation-based flash rate in each grid cell matches the average of the monthly total NLDN flash rate, where the latter is obtained by multiplying the detection-efficiency-adjusted CG flash rate by *Z* + 1, where *Z* is the climatological IC / CG ratio from Boccippio et al. (2000). This scheme, even though it is constrained by NLDN data, depends on the upstream convective precipitation predicted by the meteorological model that may be resolution dependent and generally shows low skill and large regional variations (e.g., Casati et al., 2008). With the availability of NLDN lightning flash data, an algorithm is implemented to estimate hourly LNO production from NLDN lightning flash data, avoiding the dependence on the presence of convective precipitation in the model. For modeling exercises where the observed lightning flashes are not available (e.g., real-time air quality forecasts and past- or future-year projection studies), different options are needed to provide the LNO estimates. An LNO parameterization scheme is developed based on the relationship between the observed NLDN lightning flashes and modeled convective precipitation from a set of Weather Research and Forecasting (WRF) model simulations (the model used to create meteorological inputs for CMAQ) of 2002 to 2014 over the continental United States.

In this paper, we present the updates and development of the LNO module that was released in CMAQ version 5.2 in June 2017 and present a preliminary assessment of the spatial and temporal distribution of LNO columns in the existing (mNLDN), updated (hNLDN), and newly developed (pNLDN) schemes. In a follow-on paper, a comprehensive evaluation of model performance with the various schemes will be presented.

Section 2 of this paper provides descriptions of the data and model configurations. Section 3 describes the existing and updated LNO schemes in CMAQ that are based on the NLDN data. Section 4 presents an analysis of the historical relationship between NLDN lightning flashes and model-predicted convective precipitation. Section 5 provides the derivation of the parameterization scheme based on the analysis in Sect. 4. Section 6 is the assessment of the mNLDN, hNLDN, and pNLDN schemes on their production of total LNO columns. We conclude this study in Sect. 7 with discussions.

## 2 Data source and model configuration

### 2.1 NLDN data

The observed lightning activity data were obtained from the National Lightning Detection Network (NLDN) (Orville, 2008). The raw CG flashes were gridded onto the model horizontal grid cells hourly for use in the hNLDN scheme and then aggregated into monthly mean values for use in the mNLDN scheme. The NLDN CG flashes have a detection efficiency of 90%–95% and a location accuracy of approximately 500 m. The detection efficiency for NLDN IC flashes is lower and more variable (Zhu et al., 2016), so the climatological IC / CG ratio developed by Boccippio et al. (2000) is used to quantify LNO production by IC flashes.

### 2.2 Model configurations

The meteorological fields used in developing the LNO schemes are provided by WRF model simulations (Stamarock and Klemp, 2008). The WRF output fields were processed using the Meteorology-Chemistry Interface Processor (MCIP) to provide input for the CMAQ modeling system (Otte and Pleim, 2010). We leveraged on the archived WRF simulations from 2002 to 2014 to derive the regression-based scheme (pNLDN). The archived meteorological outputs were generated from three WRF versions: version 3.4 for 2002 to 2005, version 3.7 for 2006 to 2013, and version 3.8 for 2014.

NO is the direct product of lightning flashes, and after release a large portion of it can be quickly turned into NO_2_ by reacting with O_3_ and other species in the atmosphere. Under most circumstances, NO and NO_2_ coexist in chemical and/or photochemical equilibrium, so lightning-produced nitrogen oxides are generally referred to as LNO_*x*_. But only NO is involved in the actual implementation of the schemes in CMAQ. We, hereafter, refer to all the schemes as LNO schemes. All the LNO schemes include three steps: (1) derive or use observed lightning flashes at a grid cell, (2) translate the lightning flashes into total column lightning NO at the grid cell, and (3) distribute the total column NO among model layers based on vertical distribution algorithms. After the lightning NO is injected into the vertical layers, it is then combined with (added to) the existing NO from other emissions (both anthropogenic and biogenic sources). From there, it undergoes the same chemical and/or photochemical and physical processes as any other species do.

## 3 Description of the LNO module in CMAQ: existing schemes and updates

### 3.1 Lightning module and the existing LNO schemes

Beginning with CMAQv5.0, the LNO module contains two options for in-line (based on model simulated parameters at the run time) LNO production. The first option is an over-simplified parameterization that assumes that 1 mm h^−1^ of convective precipitation (CP) corresponds to 147 lightning flashes for a 36km× 36 km horizontal grid cell (which should be scaled for other resolutions). A preliminary analysis indicated that this scheme produced unrealistically excessive LNO during summer months (not shown). This option was removed from CMAQ in version 5.2.

**Figure 1.**
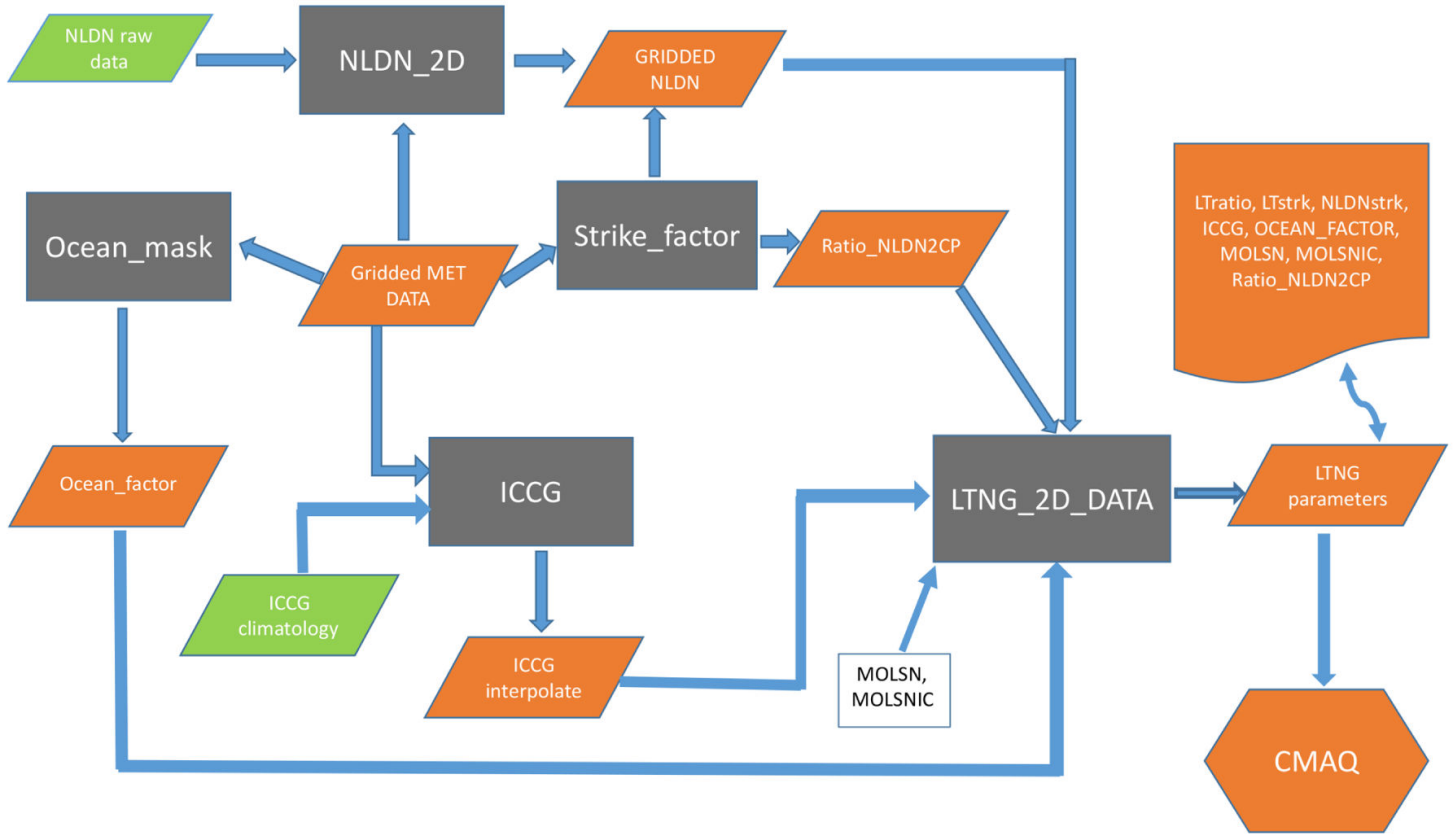
Flowchart of data preprocessing for LNO production in CMAQ for the mNLDN scheme.

The second option in CMAQv5.0 was developed by Allen et. al. (2010, 2012) and utilized monthly National Lightning Detection Network (hereafter referred to as mNLDN) flash data. In this scheme, flashes are assumed to be proportional to CP with the relationship varying locally with a two-step adjustment so that monthly average CP-based flash rates match the NLDN observations. First, a global factor (lightning yield) is applied at each grid cell to produce lightning flashes from model CP. Then, a local adjustment (LTratio) is applied at each grid cell to ensure that the local CP- and NLDN-based flash rates match. Figure 1 shows the data preprocessing for LNO production using mNLDN data in CMAQ. First, CG flashes are gridded onto the modeling grid that is specified in the model input meteorological file using the Fortran program, NLDN_2D. The output (GRIDDED NLDN) is the monthly mean lightning flash density (LFD) over the model domain in IOAPI format. Ocean_factor, Strike_factor, and ICCG are R scripts that are used to convert NLDN CG flashes to quantities that are proportional to LNO production. The Ocean_factor script ingests the land–ocean mask and indicates values of 1 for grid cells that contain land and 0.2 for grid cells that only contain ocean. A value of 0.2 is used for oceanic-grid cells because the amount of lightning produced per unit of convective rain is approximately 5 times less for marine convection than for continental convection (Christian et al., 2003). The Strike_factor script ingests the gridded NLDN CG lightning flash data and the CP values predicted by the upstream meteorological model WRF to calculate the Ratio_NLDN2CP according to the following equation:

(1) $$\text{Ratio}\_\text{NLDN2CP}=\frac{\sum_{i=1}^{\text{nT}}\sum_{j=1}^{\text{nC}}\text{NLDNflashes}}{\sum_{i=1}^{\text{nT}}\sum_{j=1}^{\text{nC}}\text{CP}},$$

where nT is the total time steps, and nC is the total grid cells. Ratio_NLDN2CP is the ratio of the monthly average total flashes over the domain to the monthly average CP over the domain, and it is used to convert the CP values to flash rates. The ICCG script interpolates the climatological IC / CG ratio (Boccippio et al., 2000) onto the model grid cells according to their geographical location and month of the year. Then the Fortran program, LTNG_2D_DATA, collects all the information generated in the prior steps plus the LNO production rate: moles NO per CG (MOSLN) and IC (MOLSNIC) flash to generate one input file (one file for each month of the year) that contains all the lightning parameters needed by the CMAQ lightning module. An additional local adjustment factor LTratio (monthly value at each grid cell) is needed to ensure that the local CP- and NLDN-based CG flash rates match.

(2) $$\text{LTratio}=\frac{\sum_{i=1}^{\text{nT}}\text{NLDNflashes}}{\sum_{i=1}^{\text{nT}}\text{CP}\times \text{Ratio}\_\text{NLDN2CP}}$$

This value is capped at 50 to avoid placing excessive amounts of lightning-NO emissions in model grid cells with much less CP than observed in an attempt to match observed monthly flash rates. Finally, the moles of NO produced per hour and grid cell are calculated in the lightning module in CMAQ as follows:

(3) CLNO=CP×Ratio_NLDN2CP×LTratio×Ocean_factor×(MOLSN + MOLSNIC×ICCG),

where CLNO is the moles of NO, and Ratio_NLDN2CP × LTratio × Ocean_factor is the lightning yield per unit CP.

### 3.2 Vertical distribution algorithm

The moles of LNO are then distributed vertically using the two-peak algorithm described in Allen et al. (2012), which is a preliminary version of the segment altitude distributions (SADs) of flash channel segments derived from Northern Alabama Lightning Mapping Array data by Koshak et al. (2014) convolved with pressure. A two-peak distribution is used because NO produced by IC flashes is centered at a higher layer of the atmosphere (350 hPa) than NO produced by CG flashes (600 hPa). Accordingly, LNO is distributed with two Gaussian normal distributions: the upper distribution has a mean pressure of 350 hPa and a standard deviation of 200 hPa, and the lower distribution has a mean pressure of 600 hPa and a standard deviation of 50 hPa. For each CMAQ layer, the pressure (*p*) is calculated as follows:

(4) p=σ×(psfc−ptop)+ptop,

where *σ* is the sigma value of the layer, psfc is the surface pressure and ptop is the pressure at the top of the model domain.

At each pressure level (*p*), the standardized Gaussian parameter (*x*) is calculated as follows:

(5) $$x=(p-\text{WMU})∕(\sqrt{2}\times \text{WSIGMA}),$$

where WMU is the mean value of the distribution (either 600 or 350 hPa) and WSIGMA is the standard deviation of the distribution (either 50 or 200 hPa).

Then the fraction of the column emissions at the pressure (*p*) is calculated by the following distribution function:

(6) Frac(x)=0.5×{1.0+SIGN(1.0,x)}×{1.0−e(−4.0×x2π)},

where SIGN is a function that produces 1.0 if *x* ≥ 0, and produces −1.0 otherwise.

At each model layer, the weighted contribution is

(7) $$W=({\text{Bottom}}_{\text{Frac}}-{\text{Top}}_{\text{Frac}})\times F1\;\;+({\text{Bottom2}}_{\text{Frac}}-{\text{Top2}}_{\text{Frac}})\times F2,$$

where *W* is the weight at a model layer, Bottom_Frac_ and Top_Frac_ are the fractional contributions calculated by Eq. (6) at the bottom and top of the model layer, respectively, for the upper distribution peak (WMU = 350 hPa, and WSIGMA = 200 hPa), and Bottom2_Frac_ and Top2_Frac_ are for the lower distribution peak (WMU = 600 hPa and WSIGMA = 50 hPa). *F*1 and *F*2 are scaling factors that control the relative contributions to *W* from the top and the bottom distributions, respectively. Ideally, *W* would match the vertical profile presented in Fig. 1 by Allen et al. (2012) and the sum of *W* at all the layers is equal to 1. In the current CMAQ configuration, *F*1 = 1 and *F*2 = 0.2.

Finally, the LNO at each layer is

(8) LTEMIS(L)=W(L)×CLNO,

where LTEMIS(*L*) is the LNO at layer *L*, *W*(*L*) is the weight at layer *L* as calculated by Eq. (7) and CLNO is the total column LNO.

### 3.3 Updates to the lightning module and the LNO production scheme

As described above, the LNO production scheme, mNLDN, calculates CLNO using scaled values of the convective precipitation. To simplify the procedure to generate LNO, in CMAQv5.2 we used the gridded hourly NLDN (hNLDN) flash data in the lightning module, which reduces Eq. (3) to

(9) CLNO=NLDNCGflashes×Ocean_factor×(MOLSN + MOLSNIC×ICCG).

NLDNCG flashes are generated using a Fortran program adapted from NLDN_2D by reading in the raw NLDN CG flashes. Ocean_factor and ICCG are the same as in Eq. (3), but the R scripts are replaced by a Fortran program to put all these parameters (including the parameters associated with regression analysis described in the next two sections) into one file as parameter input file for CMAQ. MOLSN and MOLSNIC have default values of 350 mol per flash, but they can be modified in the CMAQ run script via environment variables.

Since the hNLDN scheme directly injects LNO into the modeling grid cells based on observed lightning flashes, it is possible that desynchronization exists between LNO and other convectively transported precursor species for O_3_ production. However, when the lightning assimilation technique (Heath et al., 2016) based on the same observed lightning flashes is applied in WRF simulations, other precursor species will be forced to occur at the correct times and locations. Therefore, it is recommended that lightning assimilation be applied in WRF simulations when the hNLDN scheme is used in CMAQ to produce LNO emissions.

## 4 Examining the relationship between NLDN flashes and modeled CP

**Figure 2.**
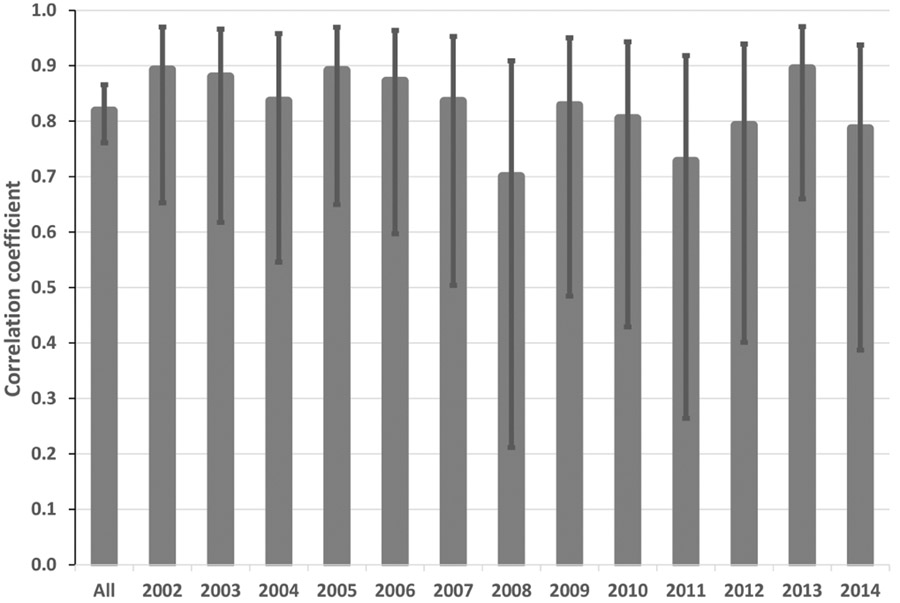
Correlation coefficients with error bars indicating the 95% confidence interval between 12 monthly mean NLDN lightning flash density and mean convective precipitation from 2002 to 2014 over the model domain. All is the correlation coefficient for all the years.

**Figure 3.**
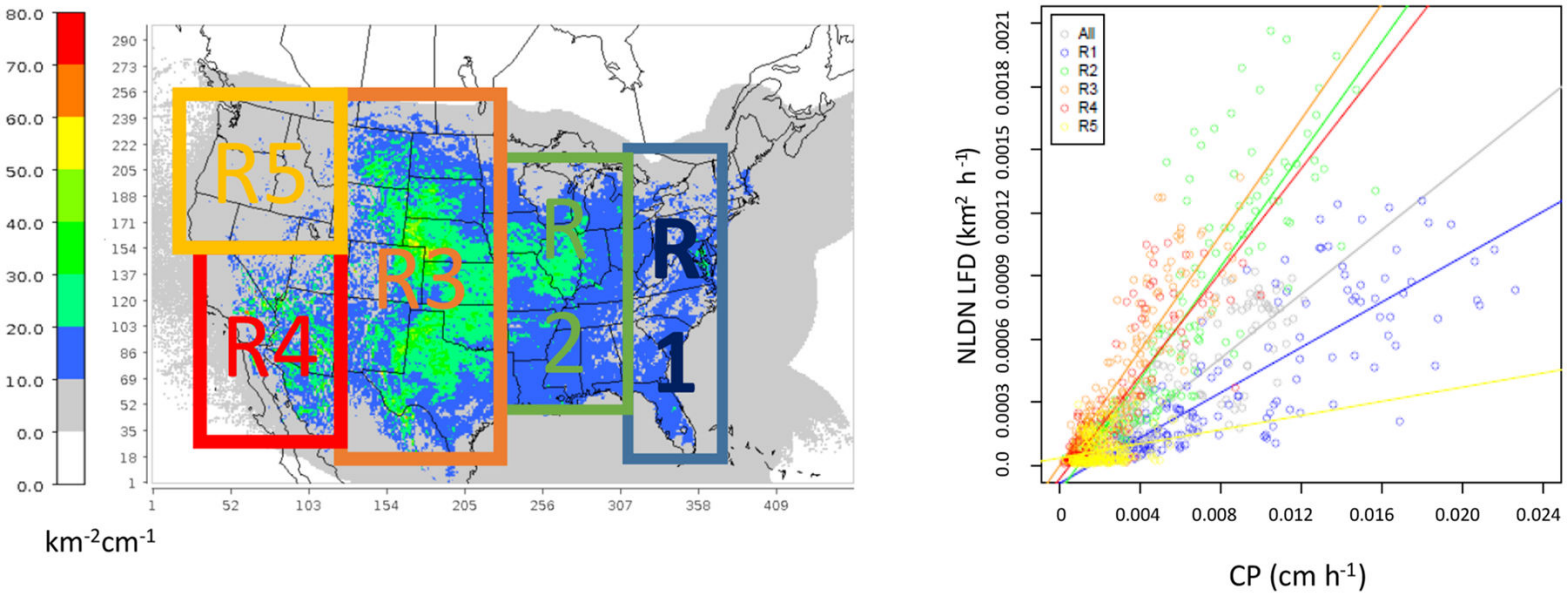
**(a)** The ratio (background) between lightning flash density and modeled convective precipitation (CP) in July (2002–2014; similar patterns for other months are not shown) and the analysis regions (R1 to R5). **(b)** Comparison of monthly mean NLDN lightning flash density (km^−2^ h^−1^) and modeled convective precipitation for the domain (All) and regions (R1 to R5) from 2002 to 2014. Each plotted pixel represents the monthly mean value: 13 (years) × 12 (months) total pixels over each region.

**Figure 4.**
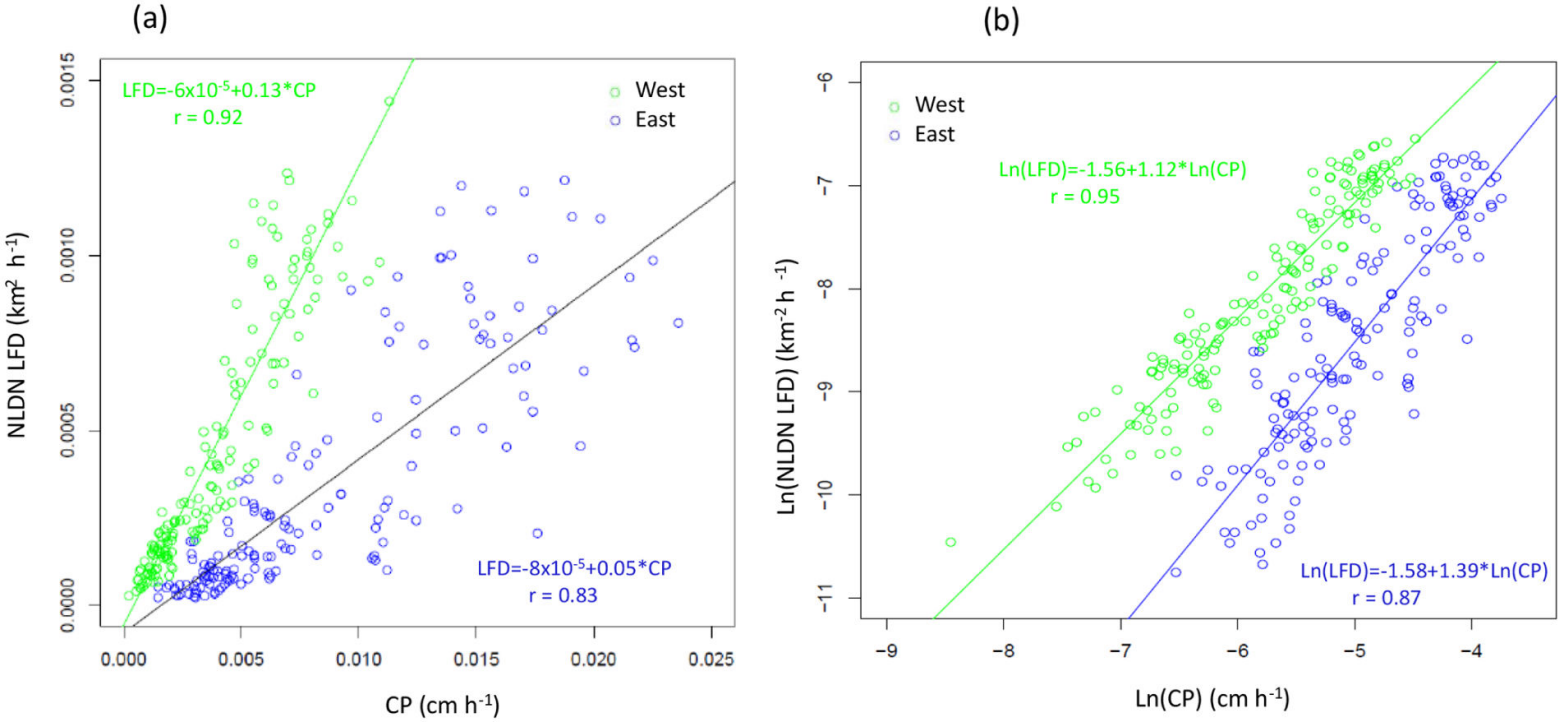
Comparison of monthly mean NLDN lightning flash density (km^−2^ h^−1^) and modeled convective precipitation for the west (green, region 1 from Fig. 3a) and east (blue, regions 2–5 in Fig. 3a) from 2002–2014 on a **(a)** linear scale and **(b)** logarithmic scale. Each plotted pixel represents the monthly mean value: 13 (years) × 12 (months) total pixels over each region.

The existing LNO production schemes in CMAQ depend heavily on CP amounts predicted by WRF. We analyzed meteorological fields generated by the WRF model simulations from 2002 to 2014 over the continental United States to examine the relationship between the observed lightning flashes and the predicted CP. Though the WRF model has evolved over a few versions (from version 3.4 to 3.8), the Kain–Fritsch (KF) convective scheme (Kain and Fritsch, 1990) was used consistently in simulations for all years. We first examined the relationship between lightning flashes, which were aggregated into hourly flash counts and gridded onto the modeling grid cells, and the modeled hourly CP from WRF over the continental United States (12 km horizontal grid spacing). The results (not shown) showed little to no correlation between the observed lightning flashes and the predicted CP, regardless of the time period examined. However, when the lightning flashes and CP were each aggregated to mean values over geographical regions (the entire modeling domain as the extreme) for each month in the time series, as shown in Fig. 2, the correlation between the two quantities was obvious. This suggests that although the model-predicted CP is not a good predictor of lightning events in space and time, it does show its skill in predicting cumulative lightning activity across geographic regions for a given month. Further analysis of the relationship indicates unique distribution patterns in space over the contiguous United States through the years. As shown in Fig. 3a and b, lightning yields per unit CP are smaller in the eastern US than in other areas confirming that the lightning yield varies regionally. The original scheme used a universal lightning yield for the entire modeling domain, while Allen et al. (2012) allowed the yield to vary locally. This analysis indicates that the yield is lowest in the east (Region 1) but similar in regions 2–5, which could be combined. Figure 4a shows the scatter plots and the corresponding linear regression equations as well as the correlation coefficients (*r*). Again, the data points over the two regions (east: region 1 and west: regions 2–5 in Fig. 3a) are distinct, and the slope (0.05) associated with the linear regression equation over the east is less than half of the value over the west (0.13), meaning that the lightning yield over the west is more than twice that over the eastern US. Further analysis reveals that better relationships exist when logarithmic translation is taken for both NLDN flashes and CP as shown in Fig. 4b; i.e., after applying the translation, the correlation coefficients increased for both the western and eastern regions.

## 5 LNO_*x*_ scheme based on the relationship between NLDN flashes and CP

**Figure 5.**
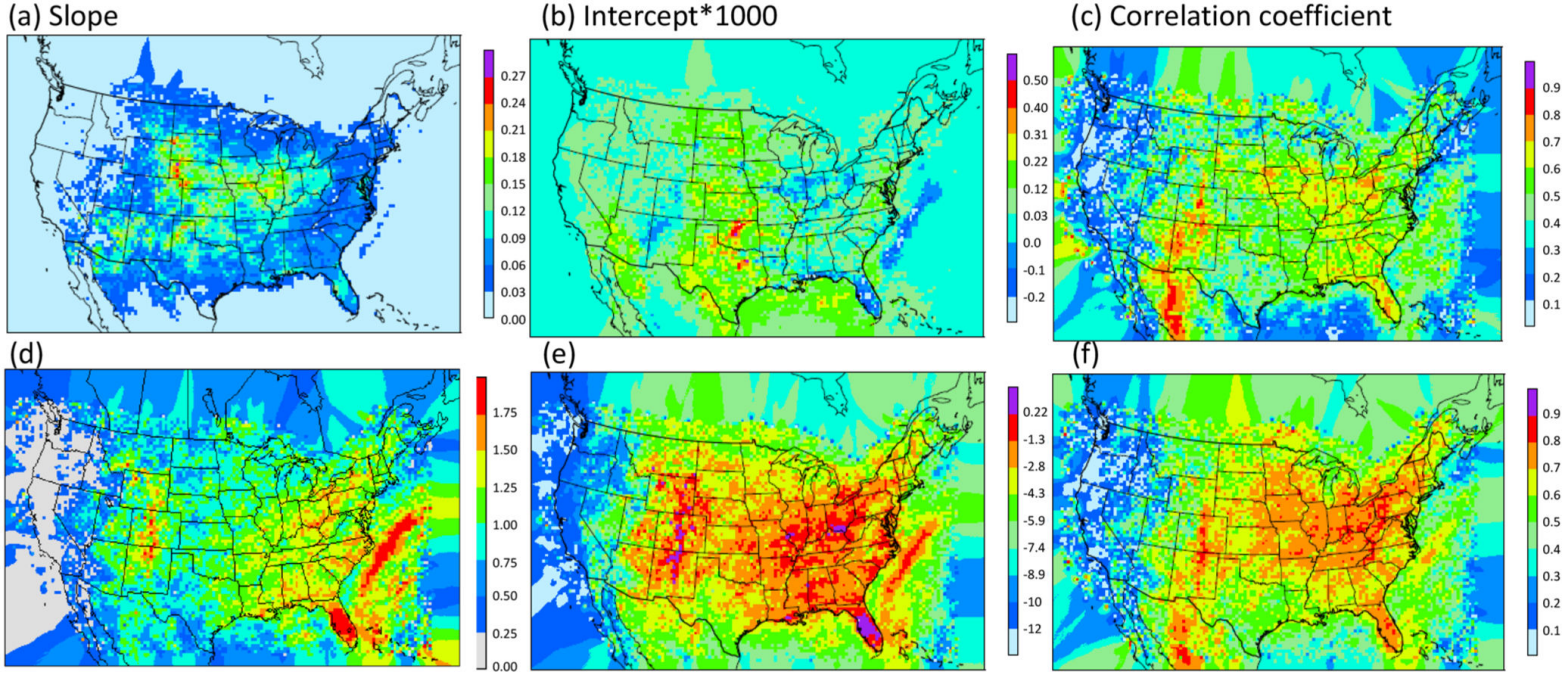
Parameters of linear **(a–c)** and logarithmic linear **(d–f)** regression parameters generated using all the data from 2002 to 2014. **(a, d)** slope, **(b, e)** intercept and **(c, f)** correlation coefficient.

Statistically, the relationship between CP rate and NLDN lightning flash rate over large regions suggests similar yields within each region. But considerable scatter still exists within each region, and the overall statistics may be dictated by certain large values. As an estimate, the most direct approach would be to use regression equations to determine LNO from CP for western US grid cells and regression equations for eastern US grid cells as shown in Fig. 4a and b. However, in addition to the concern associated with variations within a region, this direct application would also cause some practical problems: (1) the analysis regions are arbitrary, and (2) the LNO production would be spatially inconsistent with abrupt changes along the bordering grid cells separating regions. Therefore, instead of deriving regression equations using the regional data, linear (log-linear) regression equations are derived using data averaged over an area of adjacent grid cells (analogous to the derivative concept to cut regions into small areas that cover adjacent model grid cells). In areas that lack enough data points to perform the regression, data are filled using the inverse distance weighting (IDW) spatial interpolation technique (Lu and Wong, 2008). Figure 5 shows the spatial–linear (upper panel) and log-linear (lower panel) regression parameters and the correlation coefficients over patches of 3 × 3 grid cells (36 km× 36 km in area) using the data from 2002 to 2014, respectively. As shown in Fig. 5, significantly larger slope values appear over the Mountain West and Central Great Plains states indicating a greater lightning yield per unit CP over these regions than in other regions. Comparison of the two correlation coefficient maps reveals that the log-linear relationship has higher correlations over larger areas than the simple linear relationship. However, both approaches have correlation coefficients > 0.5 in regions with frequent lightning activity.

### 5.1 Stability over time

**Figure 6.**
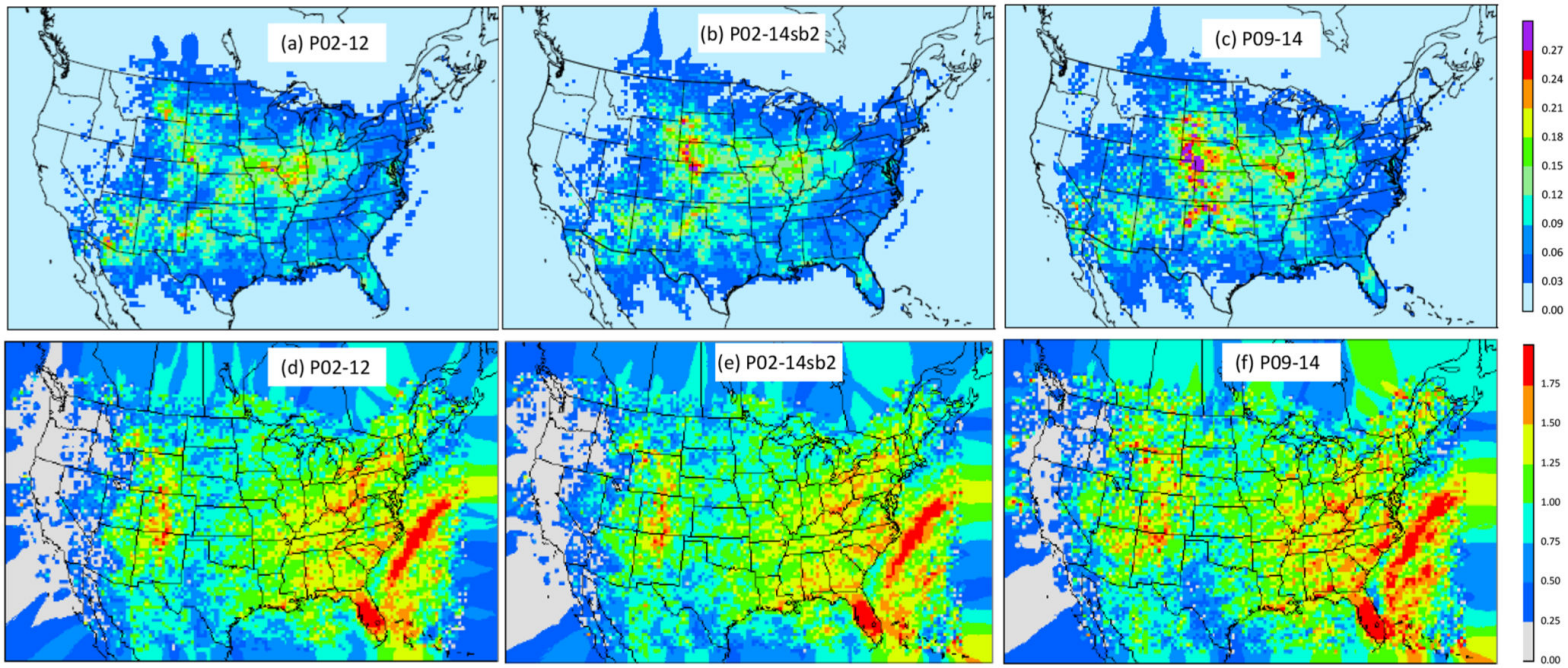
The slope maps from linear **(a–c)** and log-linear **(d–f)** regressions using data from different time periods. **(a, d)** Data from 2002 to 2012, **(b, e)** data from 2002 to 2014 excluding 2011 and 2013, and **(c, f)** data from 2009 to 2014.

A robust parameterization scheme should be relatively insensitive to the training time period. In order to test this, the lightning yield (slope of the linear and log-linear regression) was re-calculated using data from 2002 to 2012 (P02-12), 2002 to 2014 but excluding 2011 and 2013 (P02-14sb2), and 2009 to 2014 (P09-14). The results are shown in Fig. 6. As indicated in Fig. 6, the spatial patterns of slopes generated using data from different time periods for both linear (upper panel) and log-linear regressions (lower panel) are similar except that larger values are created over the Great Plains east of the mountains when the most recent years’ data (2009–2014) were used to perform the linear regression. This difference may be attributable to the evolution of the WRF model and the NLDN data (Nag et al., 2014) through the years, and it also indicates that the parameters need to be updated to include the most recent data available.

**Figure 7.**
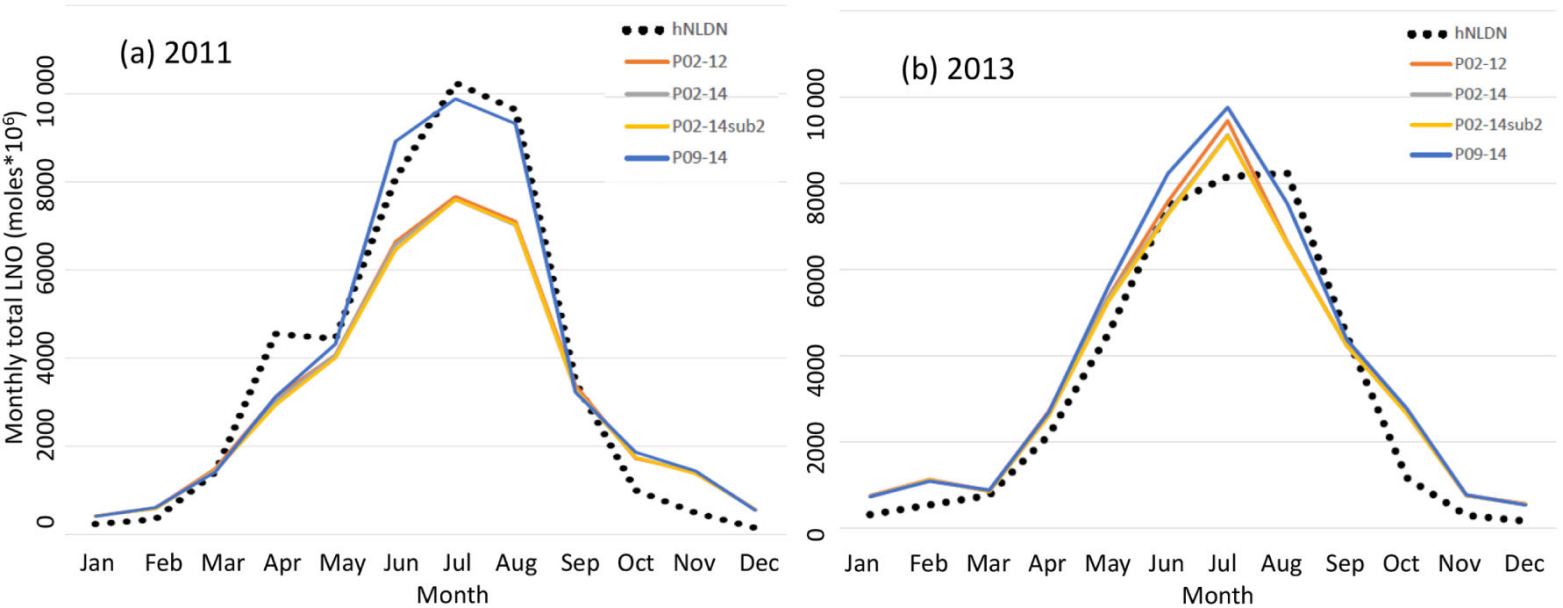
Total monthly column LNO over the model domain using parameters derived from different time periods for **(a)** 2011 and **(b)** 2013. hNLDN: LNO is produced by the hourly NLDN lightning flashes, P02-12: parameters derived using data from 2002 to 2012, P02-14: parameters derived using data from 2002 to 2014, P02-14sb2: parameters derived using data from 2002 to 2014 excluding 2011 and 2013, and P09-14: parameters derived using data from 2009 to 2014.

To test the sensitivity of LNO to the parameters derived from different time periods, Fig. 7 shows the total monthly column LNO for 2011 and 2013 generated using different set of parameters derived using linear regression from different time periods, and for comparison the LNO produced by the updated NLDN-based scheme, hNLDN, described in Sect. 2 is also included. As shown in Fig. 7a, in 2011 the parameter schemes (pNLDN) (except for P09-14) tend to underestimate LNO during summer months (June, July and August; JJA) compared with hNLDN scheme, but for 2013 (Fig. 7b) the pNLDN schemes produce both over- and underestimates of LNO during the summer months. In both years, very small differences are observed with the pNLDN scheme with parameters from different time periods except P09-14. The P09-14 parameters seem to produce the most LNO during summer months in both years making it the best to match LNO produced by hNLDN scheme in 2011, but it yields more overestimates in June and July of 2013.

### 5.2 Sensitivity to logarithmic scales

**Figure 8.**
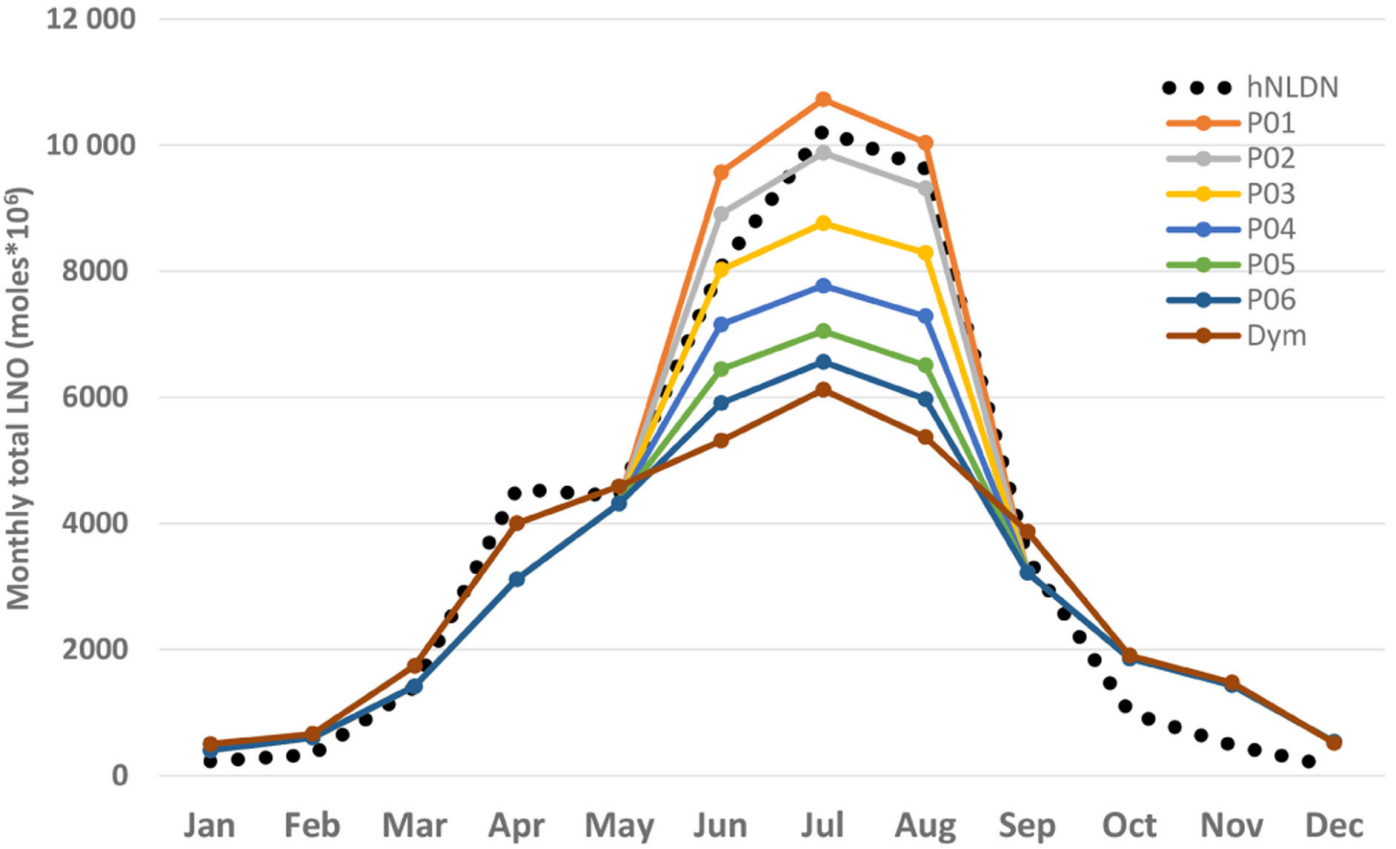
Total monthly column LNO over the model domain using different CP cutoff values during summer months in 2011. hNLDN: LNO produced by the hNLDN scheme, P01-P06: CP (cm) cutoff values from 0.01 (P01), 0.02 (P02), to 0.06 (P06). Linear regression parameters are applied when CP is less than the cutoff value and log-linear regression parameters are used otherwise. Dym is when the dynamical cutoff values are used (see text).

As discussed earlier, the log-linear regression between NLDN lightning flashes and CP produced better correlation coefficients than the simple linear regression. We also noticed, however, that if the log-scale parameters are applied to all the data, too much LNO is produced relative to the hNLDN scheme, especially during winter months when both lightning activity and convective precipitation occur less frequently. This high bias exists because the log scale tends to inflate contributions from small values when linear regression is performed after the log transformation. To test the impact of log scale on the production of LNO, we choose the summer months (JJA) in 2011 and specify a series of cutoff values for CP (cm) that is linear regression parameters are applied if CP is smaller than a specific cutoff value, and log-linear regression parameters are applied otherwise. Figure 8 shows the monthly total column LNO produced with CP cutoff values from 0.1 (P01) to 0.6 (P06) cm. As indicated in Fig. 8, the smaller the cutoff value is, the more LNO produced. When the cutoff value of 0.2 is applied, LNO production best matched those produced by hNLDN; however, the summer months in 2011 are different from other years, in that significantly more lightning flashes and convective precipitation were observed in the continental United States, especially in the east and southeast US. When the same cutoff value (0.2) is applied to other years, LNO is overestimated compared with that produced by the hNLDN scheme. For generalized application to all years, dynamic cutoff values are used with this scheme (the result is also shown in Fig. 8). Specifically, if CP is greater than the intercept value at a location from linear regression, the log-linear regression parameters are used; otherwise, the linear regression parameters are applied. This technique demonstrates acceptable results for all of the years studied.

## 6 Assessment of LNO production schemes

As a preliminary assessment of these LNO production schemes, we only investigate the distribution of column LNO in time and space; a more detailed evaluation of the impact of these schemes on air quality will be presented in a subsequent study.

**Figure 9.**
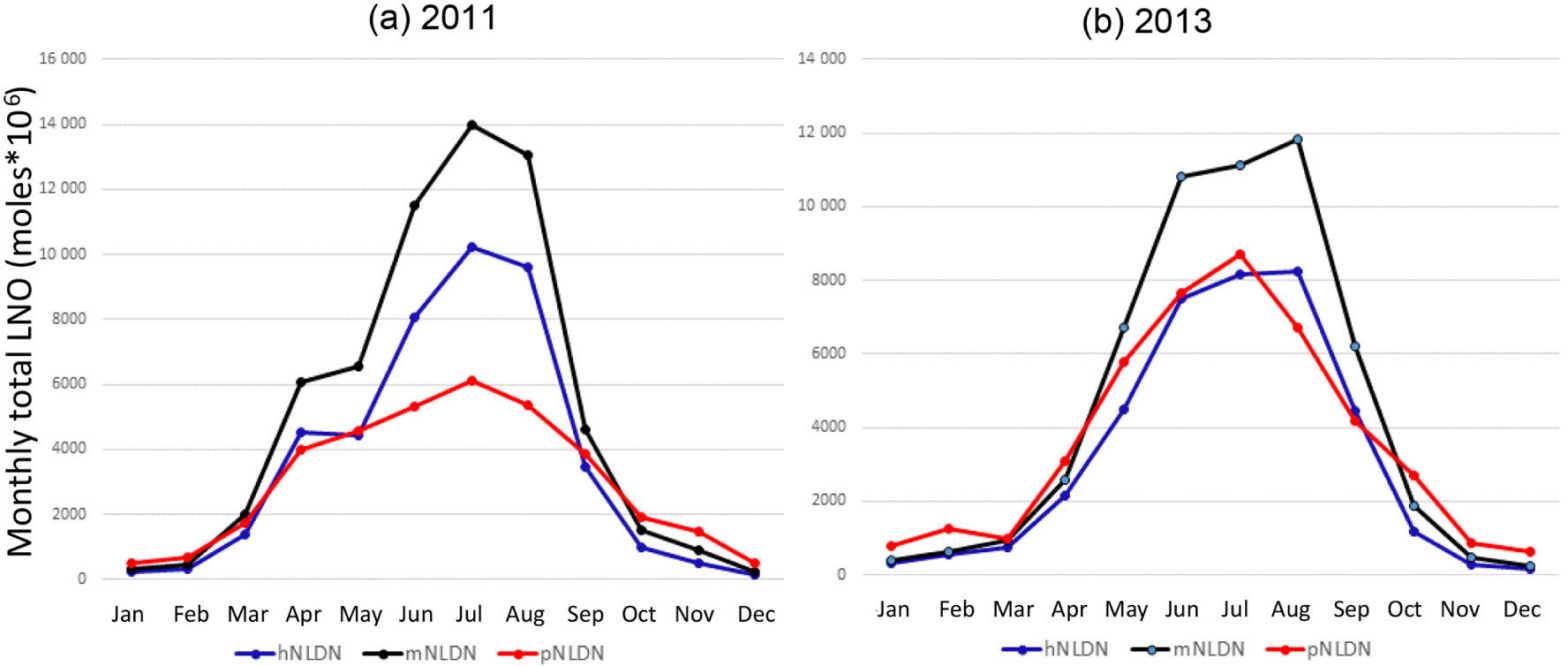
Total monthly column LNO over the model domain with different LNO production schemes for **(a)** 2011 and **(b)** 2013.

**Figure 10.**
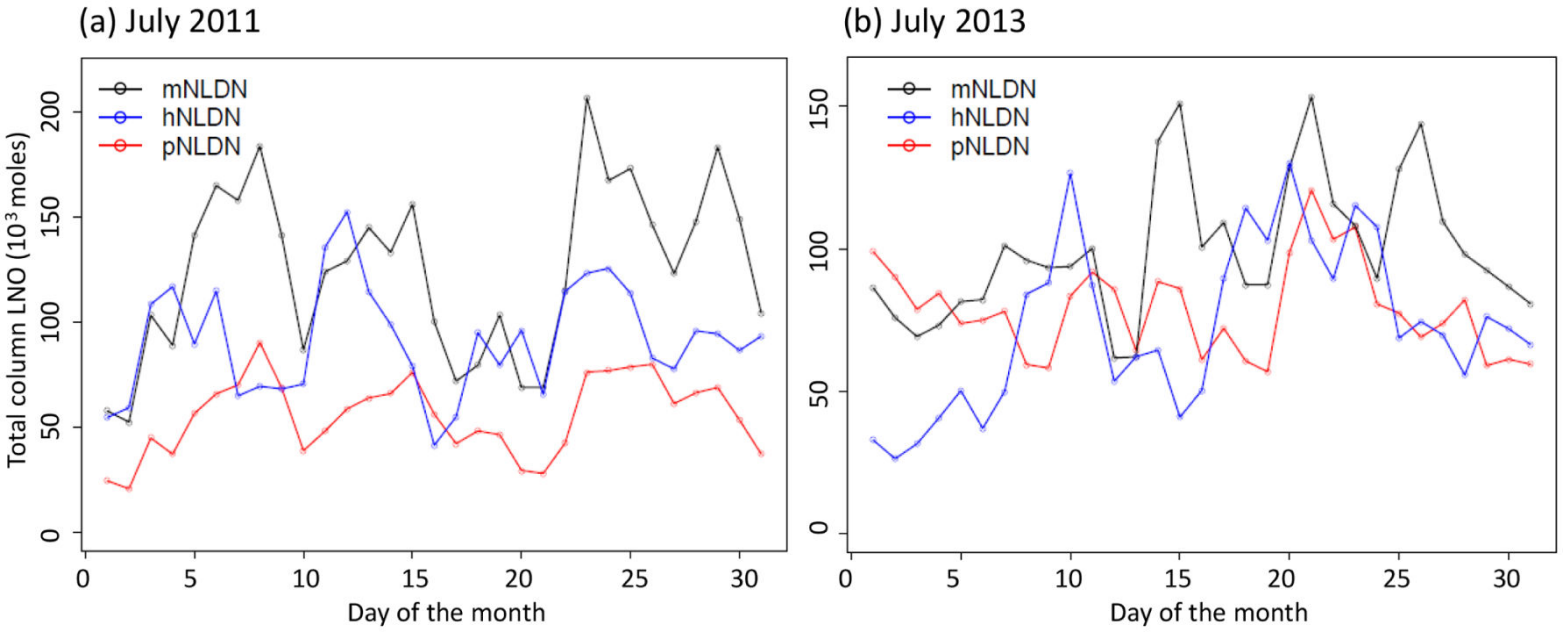
Total daily column LNO over the model domain with different LNO production schemes for **(a)** 2011 and **(b)** 2013.

Figure 9 shows the monthly total column LNO produced by the different schemes for the years 2011 and 2013. For both years, the mNLDN scheme tends to generate significantly more LNO during warm months (May–September) than the hNLDN and pNLDN schemes. Collectively during May to September, mNLDN produced about 40% (39% in 2011 and 42% in 2013) more LNO than hNLDN. The regression parameter-based scheme, pNLDN, underestimated LNO during summer months (JJA) in 2011 compared to hNLDN, but the two schemes generally agreed well in 2013. As mentioned earlier, the significant underestimation of LNO by pNLDN may be attributed to underestimated convective precipitation in WRF, which reduced the count of lightning flashes during this period. There were about 17% more lightning flashes during JJA in 2011 than the same period in 2013 over the continental United States. The relatively poor correlation coefficient between NLDN flashes and model-predicted CP values in 2011 is also evident in Fig. 2, which was the second smallest among the 13 years studied. The daily total column LNO produced by these schemes for July 2011 and July 2013 is presented in Fig. 10. Among the schemes, mNLDN produced the most LNO on most of the days in July for both years. Except for a few days, pNLDN underestimated LNO in 2011 relative to the other approaches, but in 2013 it produced comparable results to hNLDN except that it overestimated LNO on the first few days of the month. In addition, the day-to-day variance generated by pNLDN seems smaller compared with hNLDN for both years.

**Figure 11.**
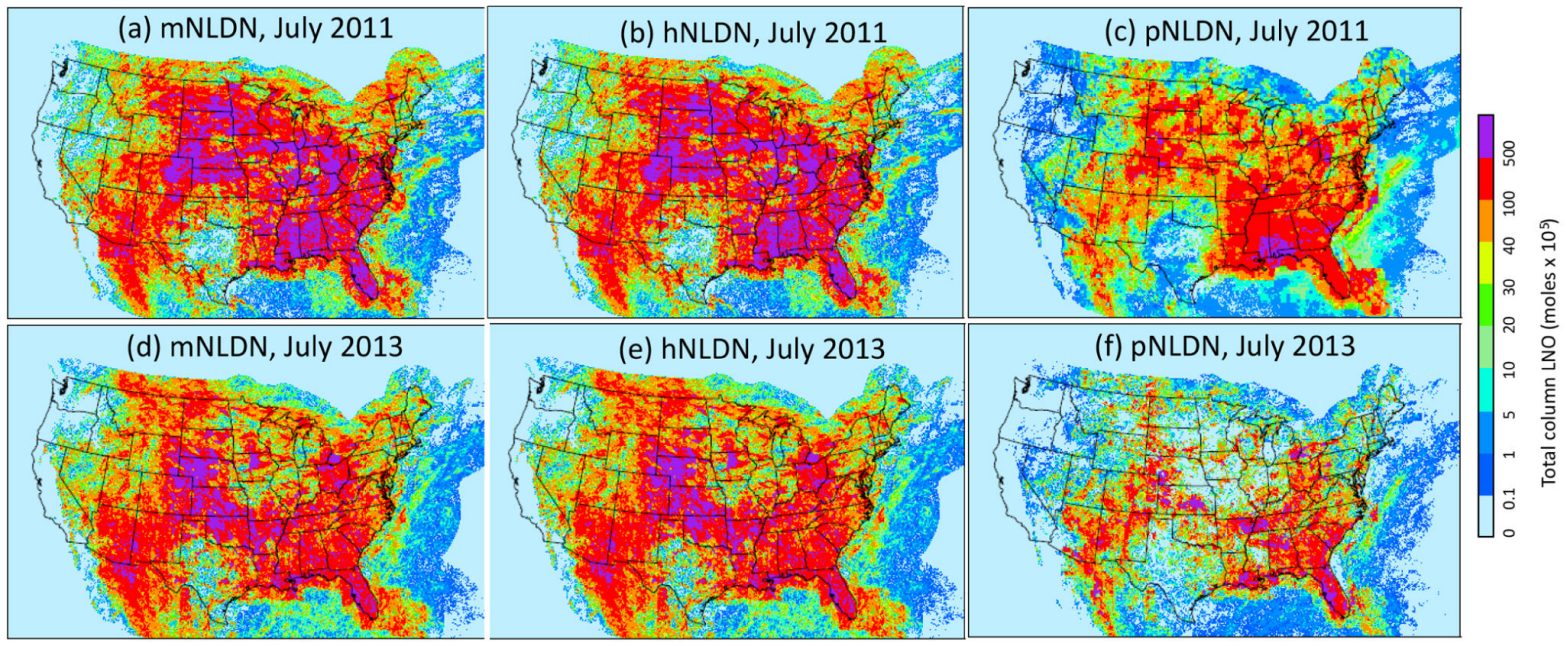
Spatial distribution of monthly column LNO with different LNO production schemes for July 2011 **(a–c)** and July 2013 **(d–f)**.

The spatial distributions of monthly total column LNO produced by each of the three schemes over the contiguous United States for July 2011 and July 2013 are presented in Fig. 11. Overall, the spatial patterns generally agree with each other for both years with pNLDN producing relatively smaller values, especially along the edges or over locations where LNO amounts are relatively small. Note that both hNLDN and mNLDN are based on the same monthly observed data, so consequently they produce similar spatial patterns. The pNLDN is derived based on the linear and log-linear regression parameters using multiple years’ historical observed data and model simulations with different versions, and it is applied to a specific period without including observations. Nevertheless, as the main intention for pNLDN to be applied is when there are no observed lightning data available (such as air quality forecasts and past or future climate simulations with similar climate conditions), it can provide a reasonable estimate for LNO comparable to that estimated by hNLDN and mNLDN.

## 7 Summary and discussions

In this study, we described the LNO production schemes in the CMAQ model’s lightning module and updated the existing monthly NLDN observation-based scheme with the current understanding and resources. For retrospective model applications, the hourly NLDN observation-based scheme, hNLDN, is expected to provide the highest-fidelity spatial–temporal LNO. If observations are not available, such as in air quality forecasts and future climate studies, the linear and log-linear regression parameter-based scheme, pNLDN, provides a spatial–temporal estimate of LNO. Note that even though the pNLDN scheme can provide LNO estimates for past or future climate studies, the spatial dependency of the relationship presented here may not hold under changing climate conditions.

Large uncertainties are still associated with each of these schemes resulting from the various assumptions common to all the LNO production schemes, e.g., the uniform NO production rate per flash, the IC / CG ratios, the difference of LNO production rates over land and ocean, and uniform vertical profiles in time and space. The regression parameter-based scheme suffers additional uncertainties resulting from the way the parameters are derived. First, the CP values were only produced by the KF convective scheme in this regression analysis. If other convective schemes are used in the upstream meteorological model, the regression relationship will differ. Spatially this scheme is only applicable to the area over which the regression analysis was performed (here, the contiguous United States). In addition, the parameters may need to be reproduced when the model resolution or version is changed or when updated observational data become available.

Lightning and LNO will remain an active research area in atmospheric sciences for the foreseeable future. For example, lightning data from Geostationary Lightning Mapper (GLM) instruments on the Geostationary Operational Environment Satellite (GOES) 16 and 17 (Goodman et al., 2013; Rudlosky et al., 2019) are now publicly available. With more observations (both at surface and in space) available, the assumptions associated with the LNO schemes will be updated to reflect the evolving understanding of LNO production in time and space. For example, Medici et al. (2017) recently updated IC / CG ratios over the contiguous United States based on the relative occurrence of CG and IC flashes over an 18.5-year period. Their study updates the Boccippio et al. (2000) climatology used in this study that employed 4-year datasets. In addition, NASA George C. Marshall Space Flight Center is updating the vertical distributions of lightning channel segments (SAD) based on 9-year North Alabama Lightning Mapping Array (NALMA) datasets (William Koshak, personal communication, 2018). In addition, the Lightning Mapping Array data could be used to obtain nominal distributions of IC and CG flashes and that information could be used to derive the scaling factors (*F*1 and *F*2) associated with the vertical LNO distribution algorithm in Eq. (7). Thus the vertical LNO distribution could be represented more accurately in time and space. When all these data are available, we will examine and adapt these updates to the lightning parameterizations and make them available in future CMAQ releases. In this paper we have developed and demonstrated a method that can now be applied to new observations as they become available.
